# Phosphorylation of phosducin-like protein BDM-1 by protein kinase 2 (CK2) is required for virulence and Gβ subunit stability in the fungal plant pathogen *Cryphonectria parasitica*

**DOI:** 10.1111/j.1365-2958.2010.07053.x

**Published:** 2010-05

**Authors:** Joanna A Salamon, Rachel Acuña, Angus L Dawe

**Affiliations:** 1 Department of Biology, New Mexico State UniversityLas Cruces, NM 88003, USA; 2 Molecular Biology Program, New Mexico State UniversityLas Cruces, NM 88003, USA

## Abstract

Phosducin-like proteins are conserved regulatory components of G-protein signalling pathways, which mediate many physiological processes. Identified throughout eukaryotic genomes, they are thought to serve as regulators of Gβγ assembly. *Cryphonectria parasitica*, a plant pathogen and causative agent of chestnut blight, contains three Gα, one Gβ, one Gγ subunits and phosducin-like protein BDM-1 that have important roles in pigmentation, sporulation and virulence. Deletion of either Gβ subunit or BDM-1 produces identical phenotypes. Additionally, we report that the Gβ subunit is not detectable in absence of BDM-1. Given that the regulatory role of phosducin-like proteins may be influenced by protein kinase 2 (CK2), we confirmed that BDM-1 is a phosphoprotein that can be targeted by CK2 *in vitro*. Mutagenesis of the five putative CK2 sites revealed that native phosphorylation likely occurs at two locations. Strains bearing a single or double serine to alanine substitutions at those sites were significantly less virulent with only minor phenotypic changes from vegetative colonies. Therefore, CK2 activity appears to mediate key signals that are required for virulence, but not for vegetative growth. Expression of selected CK2 mutants resulted in reduced accumulation of the Gβ subunit, suggesting that phosphorylation of BDM-1 influences Gβ stability.

## Introduction

Heterotrimeric GTP-binding proteins (G-proteins), consisting of three subunits Gα, Gβ and Gγ, are ubiquitous signalling components that play a well established role in the ability of eukaryotic organisms to react to environmental stimuli (Cabrera-Vera *et al.*, 2003). Functionally, the Gα protein is a GTP-ase that is active when bound to GTP. The Gβ and Gγ subunits remain as a tightly bound complex that only modulate downstream effectors when separated from the Gα partner. Upon activation by a transmembrane receptor, Gα dissociates from Gβγ, allowing both to independently regulate downstream effectors. In complex mammalian systems, G-proteins transduce a variety of hormonal, neuronal and sensory signals that may affect diverse outputs from cardiac rhythm to vision. In keeping with this complexity, the three proteins that comprise the heterotrimer (termed Gα, Gβ and Gγ; Gilman, 1987) are present in a variety of forms with as many as 20 Gα, 5 Gβ and 12 Gγ being reported (Mazzoni and Hamm, 2003).

Simpler eukaryotes, including *Saccharomyces cerevisiae, Dictyostelium discoideum*, and various filamentous fungal systems have been shown to utilize the essential features of this system, but with far fewer subunits. With the advent of genome sequence information, it has been possible to determine that Ascomycete fungal systems, for instance, generally contain three Gα subunits, accompanied by a single representative each of Gβ and Gγ. However, even with fewer subunits, the signalling processes are still associated with the control of vital behaviours such as mating (*S. cerevisiae*; Dohlman *et al.*, 1998), morphogenesis and chemotaxis (*D. discoideum*; Firtel and Chung, 2000), and asexual development (many fungi; reviewed by Lengeler *et al.*, (2000). Most importantly, G-protein signalling has also been found to be essential for the virulence of many human and plant pathogens, including *Candida albicans* (Maidan *et al.*, 2005a,b;), *Cryptococcus neoformans* (Alspaugh *et al.*, 1997), *Botrytis cinerea* (Gronover *et al.*, 2001), *Magnaporthe grisea* (Liu and Dean, 1997), *Ustilago maydis* (Regenfelder *et al.*, 1997) and, the focus of this study, *Cryphonectria parasitica* (Gao and Nuss, 1996).

*Cryphonectria parasitica*, a filamentous fungal plant pathogen of the phylum Ascomycota, is the causative agent of chestnut blight. First observed in the USA in the early part of the 20th century (Merkel, 1906), the fungus rapidly spread throughout the natural range of *Castanea dentata*, the American chestnut, resulting in the near-eradication of this species. However, hypovirulent strains of *C. parasitica* were isolated (Grente, 1965) that were subsequently shown to contain cytoplasmically transmissable factors (Grente and Sauret, 1969). These were later recognized as mycoviruses and placed in a new family, the *Hypoviridae* (Hillman *et al.*, 1995). Intriguingly, correlations have been noted between hypovirus-infected strains and those for which G-protein signalling components have been deleted. These correlations extend from phenotypic observations (Gao and Nuss, 1996; Kasahara and Nuss, 1997) to expression-level analyses by microarrays (Dawe *et al.*, 2004) and suggested that contributions to the hypovirus-infected phenotype of the fungal mycelium may arise from a partially suppressed G-protein signalling response (reviewed by Nuss, 2005).

Known modulators of the downstream signalling that stems from the Gβγ complex include the family of phosducin and phosducin-like proteins (PhLPs). These have been shown to be functionally related to Gβγ complexes in mammalian systems (Lee *et al.*, 1987; Kuo *et al.*, 1989; Miles *et al.*, 1993; Schulz, 2001), teleost fish (Kobayashi *et al.*, 2004), *S. cerevisiae* (Flanary *et al.*, 2000) and *Dictyostelium discoideum* (Knol *et al.*, 2005). In higher eukaryotes, this activity has been suggested to negatively regulate the downstream effects of the Gβγ complex by sequestering these subunits (Schulz, 2001). In *D. discoideum*, however, a PhLP is essential for proper Gβγ dimer formation (Knol *et al.*, 2005). In *C. parasitica*, the deletion of *bdm-1* results in a phenotype almost identical to that of a strain lacking the Gβ subunit CPGB-1 (Kasahara *et al.*, 2000), suggesting that BDM-1 is required for correct Gβ function, and by inference, the Gβγ complex. These lines of evidence point to a positive regulatory function that has been supported by evidence that PhLP is required the sequential assembly of Gβγ dimer in conjunction with cytosolic chaperonin complex (CCT; Lukov *et al.*, 2006). In this study, the authors demonstrated that, for PhLP to perform this function, it must be phosphorylated by protein kinase 2 (CK2). In the absence of this phosphorylation, proper assembly of the Gβγ complex is blocked.

In order to better understand the potential regulation of Gβγ activity through modulation by PhLP in fungi, we have analysed the requirements for function of the BDM-1 protein from *C. parasitica.* We report that BDM-1 is a phosphoprotein that can be a target for CK2 activity and that this phosphorylation is functionally relevant. We have observed that elimination of phosphorylation at these sites caused a significant reduction in virulence and the quantity of both Gβ and BDM-1 proteins, suggesting a role for BDM-1 in protein stability that may be related to its function in mammalian systems as a potential regulator of the CCT complex (Lukov *et al.*, 2005; 2006;).

## Results

### BDM-1 is required for Gβ subunit stability

**Fig. 1:**
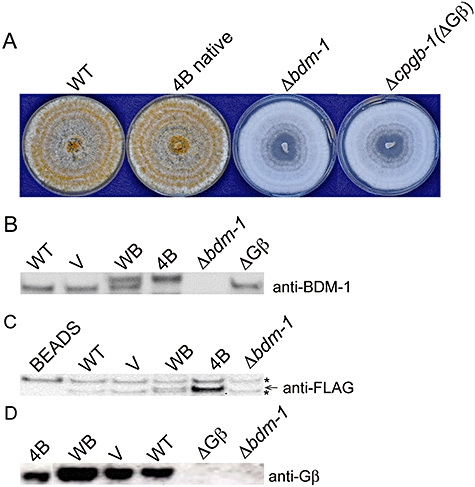
Absence of BDM-1 causes a loss of CPGB-1 protein. A. N-terminal FLAG-BDM-1 complements Δ*bdm-1*. B. Some 25 µg of total protein probed with anti-BDM-1. C. The recombinant proteins from total lysates immunoprecipitated with ANTI-FLAG M2-agarose beads and eluted. The recovered proteins separated by SDS-PAGE were probed with anti-FLAG (Sigma). BEADS refers to ANTI-FLAG M2-agarose beads not exposed to protein lysates but eluted with the loading buffer and used as a control. Asterisks represent non-specific bands, the upper arising from the beads and the lower from cross-reactivity seen in all lysates. The arrow indicates the location of FLAG-tagged BDM-1. The remaining lanes follow the strain key. D. The absence of Gβ subunit (CPGB-1) in Δ*bdm-1* lysates. Some 25 µg of total protein probed with anti-CPGB-1. Strain key: WT (wild-type, EP155); 4B native (Δ*bdm1* complemented by FLAG-BDM-1 driven by native *bdm-1* promoter); Δ*bdm1* (*bdm-1* null mutant); Δ*cpgb-1* (*cpgb-1* null mutant, ΔGβ); V (transformant with vector alone); WB (WT transformant with FLAG-BDM-1 driven by *gpd* promoter); 4B (Δ*bdm1* complemented by FLAG-BDM-1 driven by *gpd* promoter).

To analyse the post-translational modifications of BDM-1 we have tagged the N-terminus of *bdm-1* gene with FLAG peptide using primers listed in Table 1. Resulting constructs were subcloned into the two different integrating vectors listed in Table 2. pCPXNBn1 contained the constitutive *gpd* promoter and pBC6HC1 the native *bdm-1* promoter respectively. Integrated into the *C. parasitica* genome by transformation of Δ*bdm-1* and wild-type (WT) spheroplasts, both constructs rescued the distinct phenotype of Δ*bdm-1* (Fig. 1A). Western blot analysis confirmed the expression of the FLAG-BDM-1 protein (Fig. 1B). Total protein extracts from recombinant strains expressing FLAG-tagged BDM-1 were immunoprecipitated with ANTI-FLAG M2-agarose beads and detected with anti-FLAG, which demonstrated enrichment for FLAG-BDM-1 from total protein lysates (Fig. 1C).

**Table 2:** List of plasmids used in this study.

Plasmid	Construct	Description/mutation
pJS-2	pCR2.1-TOPO FLAG-Bdm-1^a^	FLAG-Bdm-1 in TOPO vector
pJS-2X	pSC-A-amp/kan FLAG-Bdm-1^b^	FLAG-Bdm-1 in pSC-A vector
pJS-3	pCPXNBn1-FLAG-Bdm-1^c^	FLAG-Bdm-1 in expression vector (*gpd* promoter)
pJS-3X	pBC6HC1- FLAG-Bdm-1^d^	FLAG-Bdm-1 in expression vector (native promoter)
pJS-4	pCR2.1-TOPO FLAG-Bdm-1 (S^45^A)^c^	m1A
pJS-5	pCR2.1-TOPO FLAG-Bdm-1 (S^55^A)^c^	m2A
pJS-6	pCR2.1-TOPO FLAG-Bdm-1 (S^130^A)^c^	m3A
pJS-7	pCR2.1-TOPO FLAG-Bdm-1 (S^136^A)^c^	m4A
pJS-8	pCR2.1-TOPO FLAG-Bdm-1 (S^213^A)^c^	m5A
pJS-9	pCR2.1-TOPO FLAG-Bdm-1 (S^45^,^136^A)^c^	m14A
pJS-10	pCR2.1-TOPO FLAG-Bdm-1 (S^130^,^136^A)^c^	m34A
pJS-11	pCR2.1-TOPO FLAG-Bdm-1 (S^45,130,136^A)^c^	m134A
pJS-12	pCR2.1-TOPO FLAG-Bdm-1 (S^45,55,130,136^A)^c^	m1234A
pJS-13	pCR2.1-TOPO FLAG-Bdm-1 (S^45,55,130,136,213^A)^c^	m12345A
pJS-14	pCR2.1-TOPO FLAG-Bdm-1 (S^45^D)^c^	m1D
pJS-15	pCR2.1-TOPO FLAG-Bdm-1 (S^55^D)^c^	m2D
pJS-16	pCR2.1-TOPO FLAG-Bdm-1 (S^130^D)^c^	m3D
pJS-17	pCR2.1-TOPO FLAG-Bdm-1 (S^136^D)^c^	m4D
pJS-18	pCR2.1-TOPO FLAG-Bdm-1 (S^213^D)^c^	m5D
pJS-19	pCR2.1-TOPO FLAG-Bdm-1 (S^45^,^136^D)^c^	m14D
pJS-20	pCR2.1-TOPO FLAG-Bdm-1 (S^130^,S^136^D)^c^	m34D
pJS-21	pCR2.1-TOPO FLAG-Bdm-1 (S^45,130,136^D)^c^	m134D
pJS-22	pCR2.1-TOPO FLAG-Bdm-1 (S^45,55,130,136^D)^c^	m1234D
pJS-23	pCR2.1-TOPO FLAG-Bdm-1 (S^45,55,130,136,213^D)^c^	m12345D
pJS-24	pCR2.1-TOPO FLAG-Bdm-1 (S^55,130,136,213^D)^c^	m2345D
pJS-25	pCR2.1-TOPO myc-Gβ^a^	myc-Gβ in TOPO vector
pJS-26	pCPXNBn1- myc-Gβ^c^	myc-Gβ in expression vector

a pCR2.1-TOPO vector (Invitrogen).

b pSC-A-amp/kan vector (Stratagene).

c Constructs subcloned into the integrating vector pCPXNBn1.

d Constructs subcloned into the integrating vector pBC6HC1.

**Table 1:** Oligonucleotide primers used in this study.

Primer	Gene/function	Oligonucleotide sequence 5′→3′
JS-10F	BDM-1/m2Ala (S^55^A)	cagtaccgtgccgcaaagatcgacg
JS-12F	BDM-1/m1Ala (S^45^A)	cgccagagacaacgctgacgacgaggag
JS-14F	BDM-1/m3Ala (S^130^A)	gcgactccaagagcgctgactctgaagagc
JS-16F	BDM-1/m4Ala (S^136^A)	actctgaagagcacgccggcgatgaggacg
JS-18F	BDM-1/m5Ala (S^213^A)	gtctgaggtctgcgccctgatcgagtc
JS-20F	BDM-1/m1Asp (S^45^D)	cgccagagacaacgatgacgacgaggag
JS-22F	BDM-1/m2Asp (S^55^D)	cagtaccgtgccgacaagatcgacg
JS-24F	BDM-1/m3Asp (S^130^D)	gcgactccaagagcgatgactctgaagagc
JS-26F	BDM-1/m4Asp (S^136^D)	actctgaagagcacgacggcgatgaggacg
JS-28F	BDM-1/m5Asp (S^213^D)	gtctgaggtctgcgacctgatcgagtc
5′-FLAG	BDM-1/added FLAG at 5′ end	gatggattacaaggatgacgacgataagtctaagactgccgcccaggaagaa
3′-HindIII	BDM-1/added HindIII at 3′ end	aaaagcttcagatgatgccgtggcgcagaa
JS-30F	Gβ/added myc at 5′ end	gatggaacaaaaactcatctcagaagaggatct
JS-31R	Gβ/added HindIII at 3′ end	aaaagcttctagtacgcccagattttgagcaat
JS-45R	BDM-1/added XbaI at 3′ end	ggtctagagcatgcgttaacaagcttca

We were able to detect the BDM-1 protein in ΔGβ lysates (Fig. 1B) but contrary to a previously published observation (Kasahara *et al.*, 2000), the Gβ subunit was absent from Δ*bdm-1* lysates of *C. parasitica* prepared from liquid-grown mycelium (Fig. 1D) or from solid medium (data not shown). The difference may be attributable to the use of an entirely different antiserum for the experiments reported here. We have used a preparation of the anti-CPGB-1 antiserum prepared subsequent to the previous study, and one that was affinity purified using recombinant CPGB-1. This preparation has been successfully used to document changes in Gβ accumulation by Parsley *et al.* (2003) and Dawe *et al.* (2004) in both solid and liquid medium.

### BDM-1 is phosphoprotein and can be a target for CK2 activity *in vitro*

**Fig. 2:**
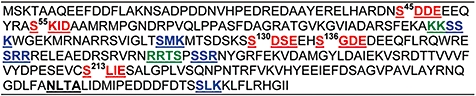
Analysis of BDM-1 amino acid sequence with PROSITE revealed putative modifications: five CK2 phosphorylation sites (red), two PKA sites (green), five PKC sites (blue), one N-glycosylation site (black).

**Fig. 3:**
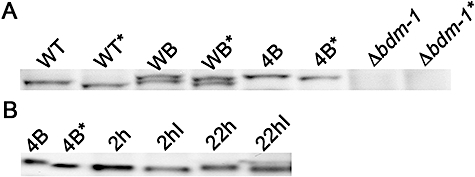
BDM-1 is a phosphoprotein that can be targeted by CK2 *in vitro*. A. Some 35 µg of total protein lysates treated with (CIP) and probed with anti-BDM-1. Phosphatase-treated proteins are indicated with *. B. Re-phosphorylation of FLAG-BDM-1 *in vitro.* Bound FLAG-BDM-1 was de-phosphorylated (*) then rephosphorylated in the presence and absence of CK2 inhibitor DMAT (I) for 2 or 22 h as indicated. Blots probed with anti-BDM-1. Lysate key: WT (wild-type, EP155); WB (WT transformant with FLAG-BDM-1 driven by *gpd* promoter); 4B (Δ*bdm1* complemented by FLAG-BDM-1 driven by *gpd* promoter); Δ*bdm1* (*bdm-1* null mutant).

Post-translational modification motifs were predicted by PROSITE (http://ca.expasy.org/prosite/), which revealed putative sites that included five for CK2, two for protein kinase A (PKA), five for protein kinase C (PKC) and one site for N-glycosylation (Fig. 2). To determine whether there were any physiologically targeted phosphorylation sites within the BDM-1 protein, we treated whole-cell lysates with Calf Intestinal Alkaline Phosphatase (CIP) *in vitro* (Fig. 3A). Based on previous reports describing mammalian PhLP-1 as target for CK2 (Carter *et al.*, 2004; Lukov *et al.*, 2005; 2006;), we tested whether BDM-1 could also be a potential substrate of CK2 phosphorylation. By incubating BDM-1 bound to FLAG beads with protein lysates from *C. parasitica* strain EP155 (WT), the migration pattern of BDM-1 was restored to that of BDM-1 prior to CIP treatment (Fig. 3B). This process was inhibited by the presence of 2-dimethylamino-4, 5, 6, 7-tetrabromo-1H-benzimidazole (DMAT), the most potent and specific CK2 inhibitor currently known (Pagano *et al.*, 2004), indicating that BDM-1 can be a target for CK2 activity *in vitro*.

### Mutational analysis of putative CK2 phosphorylation sites

Since we demonstrated that BDM-1 is post-translationally modified via CK2 activity, we have investigated the nature of this modification in more detail. Previously, QTOF mass spectrometry was used to examine the modifications of PhLP (Carter *et al.*, 2004). Despite the success of our immunoprecipitation (Fig. 1), however, it was not possible to recover sufficient quantities of FLAG-BDM-1 for mass spectrometry analysis. Therefore, to assess which of the CK2 sites identified by PROSITE are phosphorylated in physiological state of native BDM-1 protein, we engineered a series of mutations with single or multiple serine residues substituted by either alanine (unable to be targeted by CK2) or aspartic acid (considered to mimic constitutive phosphorylation; Kaufman *et al.*, 1989). Constructs listed in Table 2 were transformed into Δ*bdm-1* spheroplasts, and isolated total protein lysates were examined by Western blot for the migration rates of the FLAG-BDM-1 phosphorylation mutants in comparison to that of unmodified FLAG-BDM-1. Migration patterns were predicted to be altered by the mutagenesis as follows: (i) for an unmodified serine, substitution with alanine would cause no change in migration rate but substitution by aspartic acid would retard mobility; (ii) for a modified serine, substitution with alanine would cause an increase in migration rate, whereas an aspartatic acid substitution would not change the mobility. Lastly, treatment with CIP would remove only phosphate moieties and not alter any mobility effects induced by aspartic acid substitution.

**Fig. 4:**
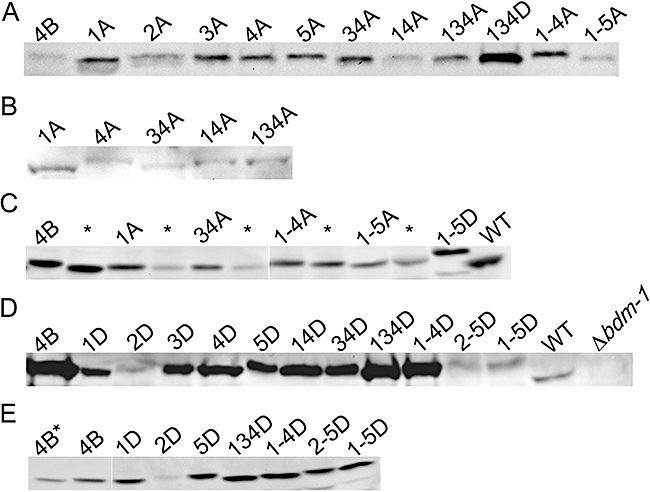
A–E. Altered migration of BDM-1 phosphorylation site mutants. Some 25 µg of total protein isolated from the BDM-1 phosphorylation site mutant expressing strains probed with anti-BDM-1. CK2 phosphorylation-site mutants (m) are labelled with the number corresponding to a single or combination of mutated sites, and letter A (Ser to Ala) or D (Ser to Asp) representing substitution type (see Table 2). CIP de-phosphorylated lysates are loaded to the right of each untreated sample and indicated with ‘*’. Lysate key: WT (wild-type, EP155); 4B (Δ*bdm1* complemented by FLAG-BDM-1 driven by *gpd* promoter); Δ*bdm1* (*bdm-1* null mutant). For clarity, mutants in consecutive sites have been truncated, e.g. m12345A is labelled m1-5A.

Single alanine substitution mutants m2A, m3A, m4A and m5A (the numbers refer to the order of the consensus CK2 phosphorylation sites along BDM-1 sequence, as identified in Fig. 2) migrated at the same rate as non-mutated FLAG-BDM-1, whereas mutant m1A exhibited overall fastest mobility (Fig. 4A). The level of BDM-1 protein in the m2A mutant was barely detectable; therefore, in order to compare its migration with the subsequent alanine mutants we increased the loading by eightfold (Fig. 4A).

Although mobility of multiple mutants m14A, m134A and m12345A was similarly increased compared with single substitutions at positions m2A, m3A, m4A, m5A as well as unchanged FLAG-BDM-1, double mutant m34A migrated even faster. Nevertheless, its mobility rate was still slightly slower than mobility of the m1A mutant (Fig. 4B). Furthermore, treatment of whole-cell lysate expressing m1A mutant with CIP caused an additional slight increase in migration equal to that of de-phosphorylated FLAG-BDM-1, whereas the same treatment of m34A lysate caused an increase in mobility equivalent to that of m1A (Fig. 4C). Intriguingly, quadruple alanine substitution mutant m1234A migrated with the same rate as intact FLAG-BDM-1 while the additional mutation at position m5 or treatment with CIP (Fig. 4C) caused further shift in protein mobility equal to that observed in mutant m12345A (Fig. 4C). Further CIP treatment of whole-cell lysate expressing mutant m12345A had no effect on its migration rate (Fig. 4C).

Analogous to the alanine substitution, aspartate mutant m1D migrated with the highest rate compared with the unaltered FLAG-BDM-1 and remaining aspartate mutants (Fig. 4D). Single and multiple substitution mutants: m2D, m3D, m4D, m5D, m14D, m34D and m1234D demonstrated equal mobility, equivalent to migration rate of intact FLAG-BDM-1 (Fig. 4D). Surprisingly, we detected increased mobility of the triple substitution mutant m134D in comparison to single aspartate mutants (Fig. 4D and E). However, mutant m134D migrated with the same rate when compared with intact FLAG-BDM-1, m134A and m12345A (Fig. 4A). Mutants containing aspartate substitutions at positions m2345D and m12345D displayed equally decreased migration rate, slower than intact FLAG-BDM-1 (Fig. 4D and E). Similarly to mutant m2A, levels of FLAG-BDM-1 protein detected in lysates prepared from the strain bearing an aspartate substitution at position m2 were greatly reduced (Fig. 4D and E).

### Loss of virulence of selected CK2 site mutants

**Fig. 5:**
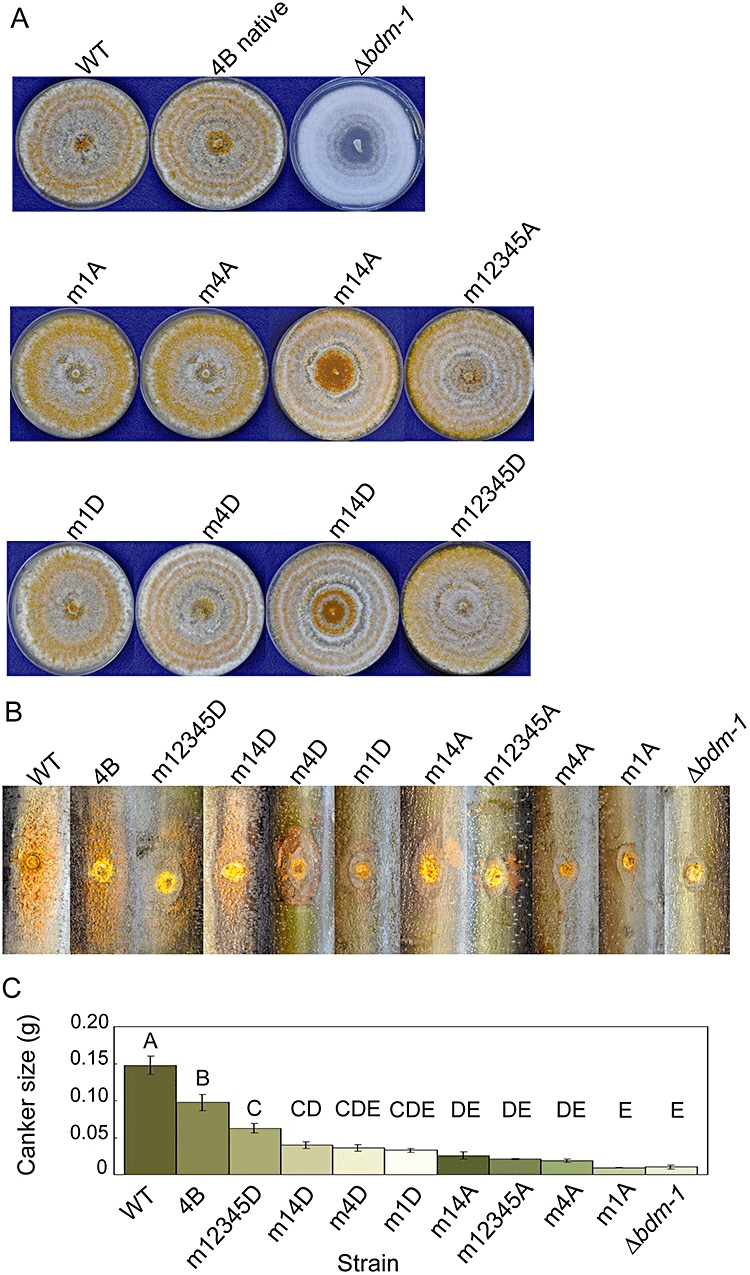
Loss of virulence of selected BDM-1 phosphorylation site mutants. A. CK2 mutants complement Δ*bdm-1* with only minor phenotypic changes. B. Representative virulence assays of CK2 mutants on dormant chestnut stems carried out for 21 days. C. Graphical representation of the virulence assay using Tukey–Kramer HSD test. *y*-axis: canker size (g weight of canker outline); *x*-axis: tested strain. Levels not connected by the same letter are significantly different. Strain key: phosphorylation site mutants key as in Table 2; WT (wild-type, EP155); 4B native (Δ*bdm1* complemented by FLAG-BDM-1 driven by native *bdm-1* promoter); Δ*bdm1* (*bdm-1* null mutant).

All of the point mutations described above were capable of complementing the *Δbdm-1* phenotype almost entirely (Fig. 5A) with only minor alterations in growth rate, colony morphology, pigment and laccase production (data not shown). However, based on analysis of migration patterns of CK2 phosphorylation mutants described in Fig. 4, we assessed the biological relevance of a subset of the single and multiple mutations that represented residues most likely target by CK2 by inoculation onto dormant chestnut stems. Statistical analysis of data collected from at least four (m1-5A) and as many as six (WT) independent virulence assays allowed us to verify significant differences in virulence of a subset of the CK2 phosphorylation site mutants (Fig. 5B and C) compared with WT and the 4B strain expressing unchanged FLAG-BDM-1. This assay also highlighted a previously undetectable impairment of the FLAG-BDM-1 function that resulted in reduced virulence.

Virulence of all tested strains expressing either single or multiple serine/alanine or serine/aspartate substitutions was significantly reduced compared with WT and FLAG-tagged BDM-1. Furthermore, all strains bearing single or multiple alanine mutations grouped statistically with the *bdm-1* deletion mutant, suggesting that their ability to cause disease was significantly impaired. However, all of the aspartate substitutions grouped together at an intermediate level between FLAG-tagged BDM-1 and the *bdm-1* null mutant, although two aspartate mutants, m14D and m1D, were also grouped with the *bdm-1* deletion strain (Fig. 5C).

### Reduced accumulation of Gβ in the presence of BDM-1 alanine-substituted mutants

**Fig. 6:**
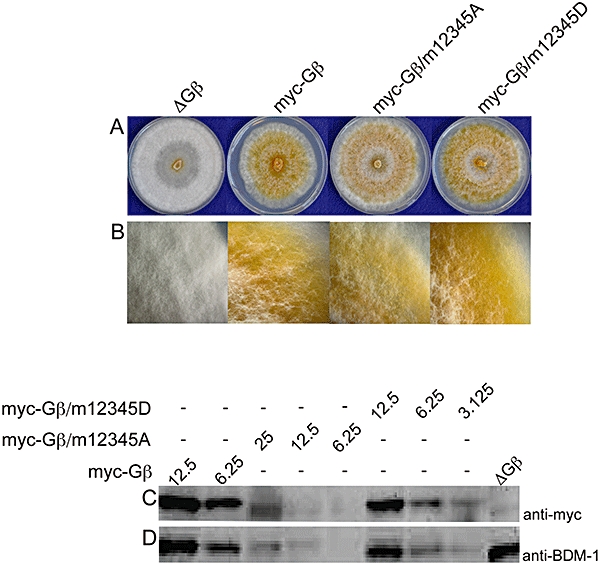
Differences in stability of Gβ and BDM-1 proteins in presence of coexpressed BDM-1 phosphorylation mutants. A. N-terminal myc-Gβ is fully functional and complements phenotypes of Δ*cpgb-1*. B. Minor phenotypic differences in heterokaryons coexpressing myc-Gβ and mutated BDM-1. The edge of growing colony revealed changes in the morphology and pigmentation of aerial hyphae between strains: Δ*cpgb-1*, myc-Gβ, myc-Gβ/m12345A and myc-Gβ/m12345D (11× magnification). C. Levels of myc-Gβ protein compared between myc-Gβ, myc-Gβ/m12345A and myc-Gβ/m12345D BDM-1 strains. Blot probed with anti-myc. D. Levels of BDM-1 protein compared between myc-Gβ, myc-Gβ/m12345A and myc-Gβ/m12345D strains. Blot probed with anti-BDM-1. Strain and lysate key: Δ*cpgb-1* (*cpgb-1* null mutant, ΔGβ); myc-Gβ (Δ*cpgb-1* complemented by N-terminally tagged myc-Gβ coexpressing unmodified BDM-1); myc-Gβ/m12345A (heterokaryon strain coexpressing myc-Gβ and mutant m12345A); myc-Gβ/m12345D (heterokaryon strain coexpressing myc-Gβ and mutant m12345D).

It has been demonstrated that nascent mammalian PhLP forms stable ternary complexes with Gβ subunit and CCT chaperonin. Furthermore, CK2-mediated phosphorylation of PhLP promotes dissociation of PhLP/Gβ dimer and allows association with the Gγ subunit (Lukov *et al.*, 2006). However, direct analysis of Gβ stability was complicated by the exhaustion of the anti-CPGB-1 used in Fig. 1. Therefore, the *C. parasitica cpgb-1* gene bearing an N-terminal myc tag was generated by PCR using primers listed in Table 1 and subcloned into the pCPX-NBn1 expression vector. This construct (Table 2), verified for sequence integrity, complemented the *Δcpgb-1* phenotype (Fig. 6A), and coupled with the Western analysis demonstrated that the myc-CPGB-1 was functional (Fig. 6C and D).

Even with the tagged Gβ, however, we lacked an additional selective marker to directly test the hypothesis that the BDM-1 phosphorylation mutants would affect CPGB-1 accumulation. To overcome this obstacle, we took advantage of the process of anastomosis to create stable heterokaryon strains expressing both tagged proteins, a process that has previously been successfully employed in *C. parasitica* for complementation studies (Kasahara *et al.*, 2000).

Due to the possible complications of the mutants at the putative CK2 target sites (as indicated by the migration patterns analysis) we used the completely substituted m12345A and m12345D variants in order to establish any potential relationship between CK2 phosphorylation and Gβ stability. Heterokaryon strains coexpressing myc-Gβ/m12345A and myc-Gβ/m12345D were first verified for presence of both genes by PCR using genomic DNA as template, then observed for distinct phenotypes in comparison to myc-Gβ expressed with non-mutated BDM-1. As with the expression of the BDM-1 mutants in the *Δbdm-1* background (Fig. 5A), the differences in phenotype were modest (Fig. 6A). We noted a slight increase in white aerial mycelium development in the strain expressing the alanine-substituted BDM-1 and myc-CPGB-1 and, contrastingly, the periphery of the strain coexpressing the aspartate-substituted BDM-1 and myc-CPGB-1 appeared to be reduced in aerial mycelium and with greater production of orange pigment (Fig. 6B).

Western blot analysis of heterokaryon strains revealed differences in the stability of myc-Gβ protein in the presence of BDM-1 phosphorylation mutants. Coexpression of m12345A caused reduced accumulation of myc-Gβ by eightfold compared with m12345D, whereas only a slight decrease of myc-Gβ protein was observed in presence of m12345D, compared with unmodified FLAG-BDM-1 (Fig. 6C). To ensure the reproducibility, levels of Gβ protein were assessed for three different sets of heterokaryons coexpressing myc-Gβ and mutated or unchanged BDM-1. Expression of mutated and unmodified FLAG-BDM-1 in heterokaryon strains was confirmed by detection with anti-BDM-1 (Fig. 6D). Consistent with accumulation of myc-Gβ, level of mutated m12345A protein was approximately four- to eightfold lower than levels of m12345D.

## Discussion

Perturbation of heterotrimeric G-protein signalling has been shown to affect virulence, pigmentation and sporulation of fungal pathogens (Lengeler *et al.*, 2000). Previous studies of *C. parasitica* have identified components of G-protein signalling, including BDM-1, a PhLP, and CPGB-1, the Gβ-subunit. Elimination of either protein reduces virulence, pigmentation and sporulation in an almost identical manner (Kasahara *et al.*, 2000). Furthermore, infection of *C. parasitica* with virulence-attenuating dsRNA viruses (hypoviruses) compromises G-protein signalling and causes changes in virulence and phenotype, with aspects that resemble those of the BDM-1 and Gβ deletion strains (Dawe and Nuss, 2001; Dawe *et al.*, 2004).

In this study, we have explored the nature of a post-translational modification of BDM-1 and its role in the stability of Gβ-subunit in the plant pathogen *C. parasitica*. Although identified and confirmed to influence G-protein signalling in *S. cerevisiae* (Flanary *et al.*, 2000), *A. nidulans* (Seo and Yu, 2006) and *C. parasitica* (Kasahara *et al.*, 2000), no further information has been available concerning the exact role for PhLPs in fungal signalling pathways. We can now confirm the functional association of BDM-1 and Gβ since the levels of Gβ protein are undetectable in the absence of BDM-1 (Fig. 1). A requirement of BDM-1 for Gβ stability explains the nearly identical phenotypes of BDM-1 and Gβ subunit knockouts and suggests that the function of BDM-1 may correlate with that noted for mammalian PhLP (Lukov *et al.*, 2005).

Evidence from mammalian systems has demonstrated that PhLPs undergo phosphorylation by CK2 that is essential for their function (Humrich *et al.*, 2003). Our observation that phosphatase-treated BDM-1 migrates faster (Fig. 3A) demonstrated that the native state of BDM-1 also involves phosphorylation at one or more sites. Most importantly, it was possible to restore migration of BDM-1 to the pretreatment level by further incubation with whole-cell lysates. However, this process could be inhibited by the addition of a specific inhibitor of CK2 (Fig. 3B). Coupled with the observation that PhLPs have been found in all branches of eukarya from fungi to humans (Willardson and Howlett, 2007), this suggests that the CK2-mediated modulation of PhLPs is a conserved process.

To determine which of the presumed CK2 phosphorylation sites are biologically relevant, we engineered a series of FLAG-BDM-1 constructs bearing mutations that either block (Ser to Ala) or mimic (Ser to Asp) CK2-mediated phosphorylation at single or multiple sites. Western blot analysis of migration patterns of the BDM-1 phosphorylation mutants (Fig. 4) allowed us to hypothesize which residues are most likely targeted by CK2 *in vivo*.

Mutants with Ser to Ala substitutions m1A, m34A, m14A, m134A and m12345A showed overall increased mobility in comparison to intact FLAG-BDM-1 (Fig. 4A), indicating that some of these residues are normally phosphorylated. Mutant m1A exhibited the fastest overall migration rate (Fig. 4B). Further shift in migration equal to that of de-phosphorylated 4B was noted after its treatment with CIP (Fig. 4C). Considering the above observations, the fact that BDM-1 could be a target for PKA and/or PKC activity (Fig. 2), and that CK2 can act in a in hierarchical manner (Hrubey and Roach, 1990), we concluded that position m1 is most likely modified by CK2. Treatment with CIP manifested an increased mobility, probably due to removal of all covalently bound phosphates that resulted from the activity of other kinases.

Although single mutants m3A and m4A migrated similarly to unmodified FLAG-BDM-1, variant m34A exhibited slightly increased migration. Thus, we suspect that either position m3 or m4 might be targeted by CK2 as well. Bearing in mind that mutants m14A, m134A and m12345A migrated with the same rate, we suggest that CK2 sites m1 and m4 are most likely phosphorylated while sites m2, m3 and m5 are not (Fig. 4A and B).

It has been reported that phosphorylation within the CK2 sites can be primed in a hierarchical fashion by previously phosphorylated residues (Roach, 1991; Salvi *et al.*, 2009). Therefore, we suggest that introduction of the phosphate at position m1 may influence the subsequent phosphorylation at remaining sites. The phenomenon of hierarchical phosphorylation within CK2 sites may serve as an explanation to the observation that the migration rate of variant m1A (in which substitution of serine by alanine inhibits additional phosphorylation at m4) is faster than predicted (Fig. 4B). Consequently, even though mutant m34A (presumably phosphorylated at position m1) migrates slightly slower than m1A (possibly due to absence of phosphate at position m4), upon treatment with CIP its migration rate is equal to that of m1A (Fig. 4C). Accordingly, mutants m14A, m134A and m12345A showed decreased migration rate compared with m34A possibly due to lack of phosphorylation at positions m1 and m4 (Fig. 4B and C). The concentration of multiple serine residues around m3 and m4 sites, along with the presence of multiple acidic residues in this region, could stimulate creation of new phosphoacceptor sites as demonstrated by Salvi *et al.* (2009) and Hrubey and Roach (1990).

Unexpectedly, migration of mutant m1234A was equal to that of unmodified FLAG-BDM-1 (Fig. 4A) and could be restored to migration rate of m12345A by treatment with CIP (Fig. 4C). A possible explanation of this observation is that simultaneous presence of alanine residues at sites m1 through 4 eliminates CK2 activity at these positions, and consequently site m5 becomes a next available target for non-hierarchical phosphorylation by CK2 or other kinase, even though the single m5A substitution suggested that m5 is not a usual target.

Comparison of migration patterns of Ser to Asp variants confirmed that sites m2 and m5 are not targeted by CK2 *in vivo* since their mobility was retarded (Fig. 4D). Interestingly, mobility of mutant m134D was faster than unmodified BDM-1 but equal to that of m134A contradicting our conclusion that site m3 is not targeted by CK2 (Fig. 4A and E). One explanation for this inconsistency is that addition of aspartate residues at sites m3 and m4 mimics phosphorylation at two mutually exclusive sites, and subsequently impairs the activity of another kinase as described for hormone-sensitive lipase (Yeaman, 1990). Since the role of serine at position m3 in hierarchical phosphorylation is not clear, it is possible that the phosphoserine itself may be involved in recognition at the active site of a secondary kinase as suggested by Roach (1991). Furthermore, predicted PKA and PKC phosphoylation sites reside within 25-amino-acid region upstream of the m3 and 35-amino-acid stretch downstream of the m4 phosphorylation sites (Fig. 2). Perhaps, despite the introduction of aspartate residues, which are considered positive determinants for CK2 phosphorylation, modified FLAG-BDM-1 becomes refractory to CK2 activity, as reported for the M8 protein of *Drosophila melanogaster* (Karandikar *et al.*, 2004).

We conclude that mutagenesis of FLAG-BDM-1 most likely altered its usual hierarchical phosphorylation scheme. Presence of the phosphate, but not the acidic substitute, may be crucial for kinase activity even without the phosphate itself participating in kinase-substrate interaction (Roach, 1991). Lastly, this may also indicate a potential role of an as-yet unidentified phosphatase participating in this scheme. Although mammalian PhLP is phosphorylated by CK2 at three of the five predicted sites (Lukov *et al.*, 2006), our current evidence indicates that CK2-mediated phosphorylation of BDM-1 *in vivo* may only occur at two sites, m1 and m4. Despite numerous Western blots performed for this study, we have never been able to observe a band detectable with BDM-1 antiserum that migrates similar to BDM-1 dephosphorylated *in vitro*. While not direct evidence, this suggests that BDM-1 is predominantly in a phosphorylated state and is supported by the observation that all of mammalian PhLP was phosphorylated by CK2 within 30 min of translation (Lukov *et al.*, 2006).

Fungal virulence is likely a complex interaction that involves a wide array of physiological and molecular determinants. Ongoing molecular studies of fungal virulence constantly uncover new genes and factors contributing to the overall pathogenicity of different fungi (Odds *et al.*, 2001). It has been previously established that BDM-1 plays a positive role in regulation of virulence of *C. parasitica* (Kasahara *et al.*, 2000). However, while grown on PDA, none of the substitutions exhibited significant effect on colony phenotype. Based on our interpretation of the gel electropheisis data, we selected a series of strains that expressed mutations at positions m1, m4, m14 and m12345 and compared their phenotypes in greater detail. As noted, these mutations appeared to complement the Δ*bdm-1* phenotype on rich medium (Fig. 5A).

When tested for virulence, however, all strains bearing these single or multiple alanine mutations were significantly impaired in their ability to cause disease, while all the aspartate substitutions were virulent at a level intermediate between FLAG-tagged BDM-1 and the *bdm-1* null mutant (Fig. 5B and C). This general trend suggests that the substitution with alanine has a larger impact on virulence than substitution with aspartic acid and supports the hypothesis that CK2 phosphorylation of BDM-1 is functionally relevant, although asparte residues in these positions are not able to fully mimic the proper physiological state. While the application of the phosphate group is critical for function under the more demanding condition of the virulence assay, removal of the phosphate likely plays an as-yet unidentified role and other mechanisms may compensate for partial function in a manner that provides for an essentially unaltered phenotype on rich growth medium.

Human PhLP-1 forms functional ternary complexes with Gβ subunits and CCT complex enabling downstream transduction of the signal, whereas disruption of PhLP-1 blocks the signalling cascade and prevents the re-association of the Gαβγ heterotrimer (Lukov *et al.*, 2006; Willardson and Howlett, 2007). It was also shown that absence of phosphorylation of mammalian PhLP at two or three consecutive serines significantly reduced Gβ or Gγ subunit that was detectable by immunoprecipitation (Lukov *et al.*, 2005).

By coexpressing tagged BDM-1 and CPGB-1 in a fused heterokaryon strain and using a serial dilution strategy, we have estimated that the accumulation of non-mutated BDM-1 and the aspartate-subsituted form are very similar (with a maximum of a twofold difference). However, the alanine-substituted mutant caused an eightfold reduction of both BDM-1 and CPGB-1 proteins (Fig. 6C and D). Therefore, phosphorylation of the PhLP BDM-1 by CK2 is important for Gβ stability. However, we cannot discern whether this is due to a reduced ability of the mutants to interact with CPGB-1, or by impairing the stability of the BDM-1 itself. This latter point may relate to the levels of BDM-1 accumulation in mutants m2A and m2D. Numerous Western blot analyses of six m2A and four m2D transformants consistently revealed an eightfold decrease in BDM-1 levels. Considering that nearly all cellular functions have been linked to CK2 activity (Pinna and Allende, 2009), and that CK2 plays central regulatory role in gene expression and protein synthesis/degradation (Pinna and Allende, 2009), we suggest that in addition to its biological relevance in virulence and Gβ stability, the CK2-dependent phosphorylation cycle of BDM-1 participates in modulating turnover of this protein.

The results presented above provide evidence for the requirement of CK2-mediated phosphorylation of BDM-1, although our current data do not allow us to state with certainty the precise phosphorylation pattern. However, we propose that, in its nascent state, BDM-1 undergoes phosphorylation by CK2, possibly at positions m1 and m4, in a hierarchical manner with m1 being targeted first. Once phosphorylated, we predict that BDM-1 is able to encourage the formation of a functional Gβγ dimer, presumably in the presence of a fungal CCT complex. This would then be followed by association with the Gγ subunit. Substitution of serine residues by alanine at positions m1 and m4 prevents phosporylation at those sites, destabilizes the process such that the signalling pathway becomes impaired to a level that is unable to fully support virulence, but does not greatly impact vegetative growth. In the complete absence of BDM-1, the assembly fails and our data would suggest that this then leads to degradation of the Gβ subunit. This model is consistent with a role for BDM-1 that is analogous to that identified for PhLP-1 in mammalian systems, in which CK2 phosphorylation was required for Gβγ assembly (Lukov *et al.*, 2006; Willardson and Howlett, 2007) and accumulation of Gβγ complex was compromised in cells depleted for PhLP-1 (Lukov *et al.*, 2005). This suggests an evolutionarily conserved mechanism for Gβγ regulation.

## Experimental procedures

### Fungal strains and growth conditions

*Cryphonectria parasitica* EP155 (ATCC 38755), Δ*cpgb-1* (Kasahara and Nuss, 1997), Δ*bdm-1* (Kasahara *et al.*, 2000) and all other strains generated for this study were maintained on potato dextrose agar (PDA; Difco) at room temperature (22–24°C) and 12 h light/dark illumination of approximately 1100 lux. Growth in liquid cultures was conducted at room temperature in potato dextrose broth (PDB; Difco) with light conditions as described above. For protein preparations, liquid cultures were grown for 3–4 days in PDB, homogenized with a Polytron PT1600E (Kinematica AG), diluted with an equal volume of fresh PDB and grown an additional 2–3 days prior to harvesting by filtration by Miracloth (EMD Biosciences). Heterokaryons were created by inoculating PDA plates with plugs of the desired precursor strains, placed approximately 2–3 mm apart. After 3 days, plugs of mycelium were recovered from the fused region and maintained on PDA.

### Protein extracts and Western blots

Protein lysates were obtained from liquid cultures essentially according to Parsley *et al.* (2003), but using a modified Protein Extraction Buffer [100 mM Tris (pH 8.0), 1 mM EDTA, 150 mM NaCl, 1% Triton X-100]. Samples were run on NuPAGE 10 or 12% Bis-Tris Gels (Invitrogen) in MOPS buffer and probed with appropriate antibodies. Images were acquired with HRP chemiluminescent detection reagents (Bio-Rad) on a Chemidoc XRS imaging system (Bio-Rad).

### Antibodies

Anti-BDM1 antiserum was generated in rabbits by Strategic Biosolutions (now Strategic Diagnostics) using purified BDM-1 isolated from *E. coli* expressing a 6-His tagged fusion protein (Qiagen QIAexpress system). The fusion protein was recovered from cell lysates using a Ni^2+^ charged HiTraP column (Amersham Biosciences). The BDM-1 binding portion was recovered from serum by passing over purified BDM-1 attached to a covalently NHS-activated Hi-trap column (Amersham). For all Western blots described below, this purified antiserum was used at 1 : 2500 dilution. Anti-Gβ (Parsley *et al.*, 2003) was kindly provided by Donald Nuss (University of Maryland Biotechnology Institute). For detection of myc tagged constructs a 1 : 1000 dilution of anti-myc –HRP (Invitrogen) antibody was used, followed by a 1 : 000 HRP-conjugated anti-mouse secondary antibody (Bio-Rad).

### N-terminal tagging, mutagenesis and immunoprecipitation

The coding sequence for the FLAG (DYKDDDDK) or myc (QGKLISQQDL) epitopes was incorporated into 5′ primers for *bdm-1* or *cpgb-1*, respectively, following the start codon while the reverse primers contained either a HindIII or XbaI restriction site. All primer sequences are detailed in Table 1. The amplified products were cloned into pCR2.1-TOPO vector (Invitrogen) or vector pSC-A-amp/kan (Stratagene) and sequenced to verify their integrity, resulting in plasmids pJS-2, pJS-2X and pJS-25 (Table 2). Digestion of plasmids pJS-2 (NotI and HindIII) and pJS-2X (NotI and XbaI) liberated fragments containing FLAG-tagged *bdm-1*, which were then subcloned into the same restriction sites of *C. parasitica* expression vectors pCPX-NBn1 (containing *gpd* promoter) and pBC6HC1 (containing native *bdm-1* promoter) resulting in plasmids pJS-3 and pJS-3X respectively. Excised from plasmid pJS-21 by restriction digest with NotI and HindIII, myc-tagged *cpgb-1* was subcloned into the expression vector pCPX-NBn1 resulting in plasmid pJS-26. Both expression vectors pCPX-NBn1 and pBC6HC1 contained a benomyl resistance gene (*ben*^r^) for selectivity.

Mutagenesis of FLAG-BDM-1 was initially performed using pJS-3 as template according to the Quick Change Site Directed Mutagenesis Kit (Stratagene) and verified by sequencing. Mutated *bdm-1* sequences were subcloned into the expression vector as described above and followed by transformation of the Δ*bdm-1* spheroplasts using the method of Churchill *et al.* (1990). For subsequent multiple mutants we used already existing constructs as their templates and added desired modifications. Single, benomyl resistant colonies were isolated from asexual spores to ensure nuclear homogeneity.

Fusion proteins contained in 35 µg of total protein lystates were bound to 20 µl of ANTI-FLAG M2-agarose beads (Sigma) for 2 h at room temperature, washed and eluted with 2× LDS buffer (Invitrogen) or processed for re-phosphorylation. Samples were run on NuPAGE 10 or 12% Bis-Tris Gels (Invitrogen) in MOPS buffer and probed with anti-BDM-1 serum followed by 1 : 2500 HRP-conjugated anti-rabbit secondary antibody (Bio-Rad).

### De-phosphorylation and Re-phosphorylation assays

Some 35 µg of clear protein lysates was incubated for 30 min at 37°C with 20 000 units of CIP (Invitrogen) then immunoprecipitated with 20 µl of ANTI-FLAG M2-agarose beads. De-phosphorylated proteins were re-phosphorylated by addition of equal amounts of WT (EP155) protein extracts in presence of 1 mM ATP (Sigma), 100 mM sodium orthovanadate (Sigma) to inhibit residual CIP activity and 20 µM specific Casein Kinase II (CK2) inhibitor 2-dimethylamino-4, 5, 6, 7-tetrabromo-1H-benzimidazole (DMAT; EMD Biosciences) where indicated. The reaction was performed at room temperature in the dark and protein eluted with 2× LDS buffer followed by separation on NuPAGE 10% Bis-Tris Gels (Invitrogen) in MOPS buffer. Protein detection was carried out as previously described.

### Virulence assay of BDM-1 phosphorylation mutants

The virulence assays were performed essentially as described by Jaynes and Elliston (1980). Dormant chestnut stems (kindly provided by W. MacDonald, West Virginia University) stored at −20°C were defrosted overnight and thoroughly cleaned with 75% ethanol. Wounds in 5 mm diameter removing the bark were made using a sterile cork borer. Fresh mycelial plugs were placed into the wounds and wrapped with parafilm to prevent desiccation. Parafilm was removed after 3 days once the plugs were well established. The experiment was carried out for a total of 21 days and relative canker size was measured by the simple method of tracing the outline of the canker edge using a piece of parafilm and weighing the resulting cut-outs. Statistical analysis of virulence data was conducted using a Tukey–Kramer HSD (honestly significant difference) test, a single-step statistical analysis that compares all possible pairs of means, and identifies which means are significantly different from one another.
